# The in-session discourse of unresolved/disorganized psychotherapy patients: An exploratory study of an attachment classification

**DOI:** 10.3389/fpsyg.2022.985685

**Published:** 2022-10-05

**Authors:** Alessandro Talia, Svenja Taubner, Madeleine Miller-Bottome, Signe Dall Muurholm, Anna Winther, Frederik Weischer Frandsen, Tine Harpøth, Antonio Onofri, Mickey T. Kongerslev, Sebastian Simonsen, Stig Poulsen, Robbie Duschinsky

**Affiliations:** ^1^Primary Care Unit, Department of Public Health and Primary Care, University of Cambridge, Cambridge, United Kingdom; ^2^Institute for Psychosocial Prevention, University of Heidelberg, Heidelberg, Germany; ^3^California Pacific Medical Center, San Francisco, CA, United States; ^4^Department of Psychology, University of Copenhagen, Copenhagen, Denmark; ^5^Stolpegaard Psychotherapy Centre Mental Health Services, Capital Region of Denmark, Gentofte, Denmark; ^6^Department of Psychiatry Mental Health Services East, Region Zealand, Roskilde, Denmark; ^7^Psychiatric Research Unit, Region Zealand, Slagelse, Denmark; ^8^School of Specialization in Psychotherapy Training School of Rome and Jesi, Rome, Italy; ^9^Department of Psychology, University of Southern Denmark, Odense, Denmark

**Keywords:** attachment, adult attachment interview, personality disorder, language, epistemic trust

## Abstract

The Unresolved/disorganized (U/d) attachment classification has generated considerable interest among clinicians. This is in part based on its empirical associations with adult mental health, parenting practices, and treatment outcomes. Despite decades of theorizing, however, we have little empirical information regarding how patients with a U/d classification assigned by accredited coders actually behave or speak in psychotherapy sessions. Here, we take a step towards bridging this gap by reporting our observations of the psychotherapy session transcripts of 40 outpatients who were independently classified as U/d on the Adult Attachment Interview (AAI), the gold standard measure of adult attachment research. These patients were extracted from a larger sample of 181 and compared to others without a U/d classification. In this paper, we discuss two different discourse styles associated with a U/d classification. Some U/d patients did not seem to sufficiently elicit the therapist’s endorsement of what they said. For example, they did not justify their claims with examples or explanations, or did not consider others’ intentions or experiences. Other U/d patients were credible, but left the listener uncertain as to the underlying point of their discourse, for example, by glaringly omitting the consequences of their experiences, or interrupting their narratives mid-way. In the discussion, we place these observations in the context of recent thinking on attachment and epistemic trust, and discuss how this study may form the basis for future quantitative studies of psychotherapy.

## Introduction

For several decades, one of the key concerns of attachment researchers has been to capture the ways in which people function in close relationships. Today, we know that the so-called “Unresolved/disorganized” (“U/d”) attachment classification is the classification most closely associated with psychological dysfunction in adults. Similar to other classifications of adult attachment, the U/d classification is assigned by analyzing an individual’s discourse style during the Adult Attachment Interview (Main et al., 2002), a semi-structured interview about early attachment relationships. A U/d classification is assigned in particular when in the AAI transcript there are indices of incoherence or confusion during recall of potentially traumatic events, such as losing a family member or instances of abuse. The classification of U/d has been shown to predict depression (Dagan et al., 2018), dissociative symptoms (Abrams et al., 2006), PTSD and personality disorders (Bakermans-Kranenburg and van Jzendoorn, 2009), as well as atypical parenting and disorganized attachment in parents’ offspring (Madigan et al., 2006; Verhage et al., 2016).

Due to evidence of its links to poor psychosocial adjustment and mental health, the U/d classification has attracted considerable interest among clinical practitioners and researchers. This interest is also due to theory proposing that a patient’s attachment-based expectations will influence the therapeutic encounter. Yet, our understanding of how the U/d classification affects the patient-therapist relationship and the clinical context more generally is surprisingly limited. Clinicians’ and researchers’ hypotheses about how patients with this classification engage in psychotherapy are still more informed by theoretical ideas about emotion dysregulation and personality dysfunction than by empirical studies (Duschinsky et al., 2021). This is unfortunate, since more evidence-based knowledge on this topic may serve as a guide for practitioners working with patients with severe mental disorders.

This paper is the first investigation of the in-therapy discourse of psychotherapy patients classified as U/d on the AAI by accredited coders. Because of the paucity of previous research in this area, we conducted an exploratory analysis into the discourse characteristics that seem to typify these patients in session. Although several speculative accounts have been advanced regarding the discourse of U/d patients in therapeutic settings (see, e.g., Wallin, 2007; Bateman and Fonagy, 2016; Farina et al., 2020), more work is needed to specify these proposals. In this regard, inductively derived accounts can be valuable for characterizing interactive processes and understanding them in context, forming the foundation for hypotheses that can then be tested in future quantitative studies.

Our study presents preliminary evidence of discourse characteristics that typify U/d patients in psychotherapy, which emerge in discussions of topics beyond traumatic events or loss. These discourse characteristics were identified with the guess and uncover method, a semi-inductive method popular in attachment research. Through this method, we compared the discourse of 40 psychotherapy patients independently coded as U/d in the AAI, or who could not be classified according to the AAI coding system (i.e., “Cannot classify”), against the discourse of 40 patients who did not receive these classifications. Here, we illustrate these characteristics with examples taken from verbatim session transcripts. This source material comes from a total sample of 181 outpatients treated with different modalities of individual psychotherapy. With this paper, we wish to expand the work of authors such as Turton et al. (2001), and Speranza et al. (2017), who reported on their observations of the discourse of speakers who were classified as U/d or Cannot Classify in the context of the AAI. Our focus will be on patients’ discourse during psychotherapy.

We will propose that the discourse characteristics of U/d patients identified by us reflect differences in how patients are able to foster the therapist’s trust in the truth and relevance of what they communicate - i.e. *epistemic trust*. Fonagy and colleagues (Fonagy et al., 2017) proposed that patients with severe personality disorders may find it difficult to experience epistemic trust in interpersonal communication. They also theorized that such difficulties may stem out of insecure and disorganized attachments. Our work in this paper draws from the additional hypothesis, advanced by Talia and colleagues (Talia et al., 2021a), that unresolved/disorganized attachment may be associated with distinct difficulties in fostering epistemic trust in others. In our analyses, some of these patients do not seem to bring evidence for what they say nor try to win the therapist sympathy or approval, whilst others do not call attention to their point of view about their experiences.

Whilst all patients in this sample have granted permission to use their clinical material, each patient’s identity is highly disguised following recommendations in Gabbard, 2000. Any details that might provide clues for recognizing patients or any other person mentioned in the transcript excerpts have been altered, including gender and age of all persons, their occupations, family relationships, professional roles, religious beliefs, cultural backgrounds, dates, places, and all other specific details of the events narrated themselves - in brief, everything that was not essential to illustrate the general discourse characteristics we wish to analyze. We are grateful to patients for their willingness for their material to be explored in this research.

We begin with a review of adult attachment classifications and how they were originally discovered through the “guess and uncover” method, before discussing the U/d classification in particular. Next, we review the methods and findings of previous research on attachment-related differences in the therapy process. We then proceed to illustrate the methods of this study and discuss its results, along with excerpts of session transcripts.

### The discovery of adult attachment classifications and the “guess and uncover” method

By the mid-80s, most developmental researchers had recognized the importance of attachment patterns and the procedure used to assess them, the Strange Situation Procedure (SSP, Duschinsky, 2020). Yet, most were convinced that attachment patterns only described behaviors relevant to the domain of infancy. Mary Main was the first to view these patterns as the only major patterns of human attachment throughout the life course. Main argued that the secure, avoidant, and resistant/ambivalent patterns of attachment represented the only three functional responses that a human can have to distress: communicate about it; keep the distress to oneself; or make it someone else’s problem (Duschinsky, 2020).

Main’s early research with the AAI was inspired by her attempt to pursue this conception of attachment patterns as lifelong individual differences (Main et al., 1985). In particular, Main wished to investigate whether differences in infants’ attachment behavior are linked to differences in representations of close relationships. Together with her colleagues, she attempted to identify criteria that would distinguish how parents of children classified as secure, avoidant, ambivalent would speak in an interview about early attachment experiences: the AAI (Main et al., 1985). Because she expected to find continuity in how attachment differences are expressed in infancy and adulthood, she introduced a research method suitable for exploring correspondences and analogues: the so-called “guess and uncover method.”

The guess and uncover method is a semi-inductive method, whose main aim is to support the discovery of observable individual differences associated with an overarching personality construct or other external criterion, for example an individual’s attachment classification. This method has been central to the development of many core attachment measures beyond the AAI (see, e.g., Main and Cassidy, 1988; George and Solomon, 2016). Yet, with the exception of a cursory mention in Main and Cassidy (1988), its primary available description is based on archival research (Duschinsky, 2020). Because a version of this method was used in this study too, we illustrate it below in some detail.

**Figure 1:**
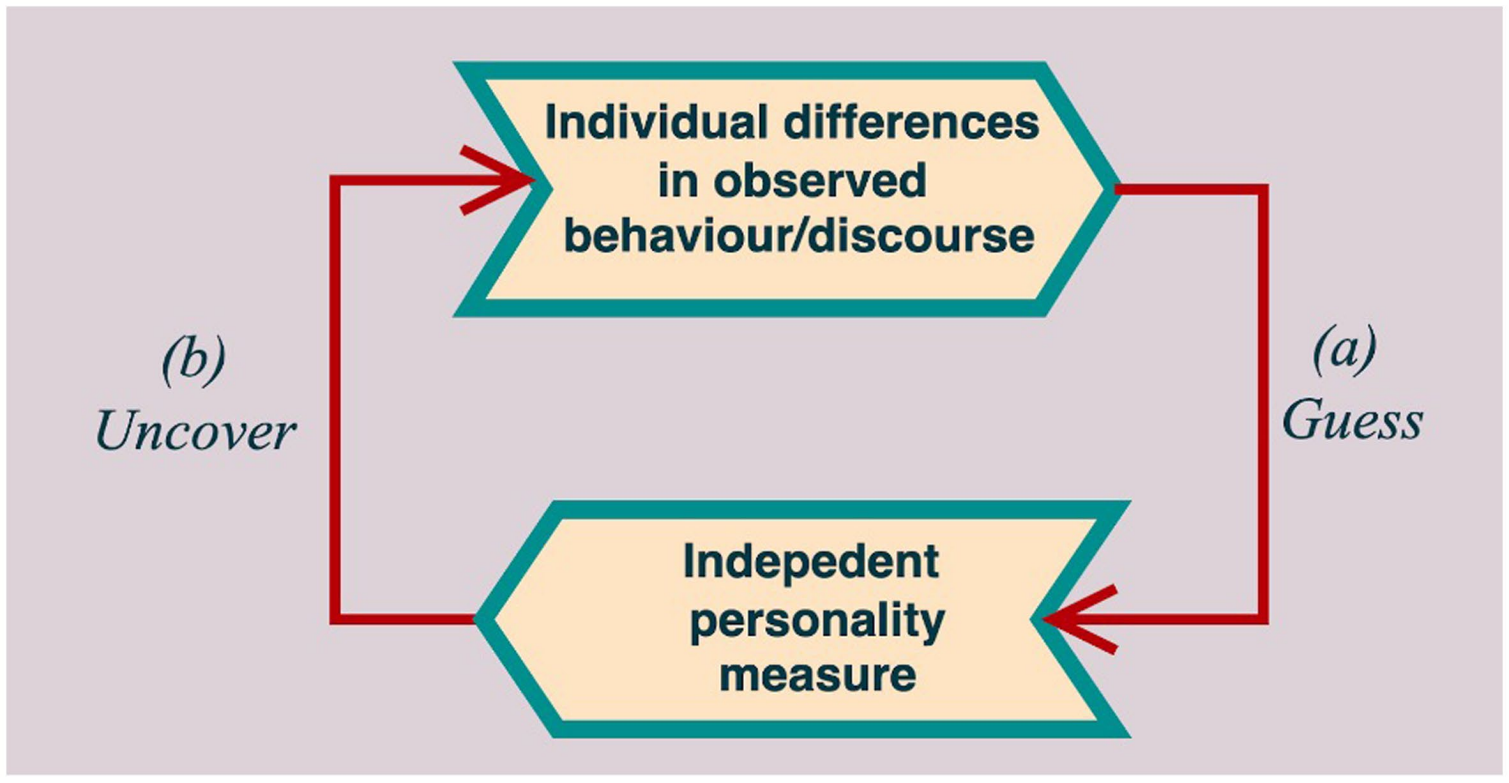
Main’s guess and uncover method.

In the first step of this method, attachment researchers make some initial conjectures about the likely attachment classification of a sample of individuals, whose behavior or discourse were observed in depth (“guess,” a, Figure 1). These conjectures are informed by *a-priori* assumptions about how attachment may manifest in the context under study. As they conduct their observations, researchers record any element of participants’ behavior or discourse that they believe to *indicate* participants’ attachment classification. They then elaborate short written descriptions for each of these indicators, in order to enable other researchers to identify them in future observations. These descriptions are the new coding system in embryonal form.

In the second step, the researchers compare their initial guesses with independently-obtained information about participants’ attachment classifications (for example, a speaker’s infant’s attachment classification, as in the original Main et al., 1985 study) and they compare such information with their initial guesses about participants’ attachment classification (b, “uncover,” Figure 1). Any error in prediction becomes an occasion for developing the system further. Indicators that lead researchers to incorrect classifications are eliminated or revised, whilst new indicators are introduced. Researchers who use the guess-and-uncover method go through the above two steps repeatedly, adapting their coding system to new samples in turn. Although this process may virtually continue indefinitely, it can stop when the indicators already discovered appear to adequately classify new cases.

Thanks to the use of the guess and uncover method for devising the AAI coding system, Main and her colleagues realized that a child’s secure attachment could be predicted by the parent’s ability to relay their early attachment experiences in a coherent, vivid manner, free of contradictions or gaps. Avoidant or resistant/ambivalent infant-caregiver attachment patterns, on the other hand, seemed to be predicted by parents’ detachment or parents’ frustration in narrating their own attachment experiences. Speakers displaying these three narrative styles were termed secure/autonomous, dismissing, and preoccupied, respectively.

Whilst the guess and uncover method has paved the way to exhilarating discoveries, it is essentially an inductive approach, the strength of which is to assist researchers in generating hypotheses rooted in empirical observation. Though valuable in the “context of discovery” (Popper, 2005), this method is not equipped for testing hypotheses at the same time. For this reason, Van IJzendoorn and Bakermans-Kranenburg (2021) have specifically argued that results produced using the guess and uncover method need to be tested in new samples before being incorporated into attachment theory and developmental science. In the case of the guess and uncover analysis that led to develop the AAI, Main and colleagues’ results have been replicated over a hundred times, confirming that parents’ AAI classifications are a robust predictor of their infants’ attachment classifications (Verhage et al., 2016).

### The unresolved/disorganized attachment classification

The concept of a ‘U/d’ state of mind with respect to attachment was introduced by Mary Main and colleagues in the late 1980s (Main and Hesse, 1990) in the context of research into the antecedents of parent-infant ‘disorganized attachment’. Disorganized attachment is a relational pattern that can be identified by observing a child’s reunion behavior with a caregiver after brief separations in the SSP (Ainsworth et al.,1978), through the simultaneous presence of apparently contradictory behaviors towards the caregiver (e.g., approach and avoidance), or signs that the child is confused or may even be frightened by the caregiver (Main and Solomon, 1986). Main and Hesse came to view attachment disorganization as an interruption or as a *dysfunctional breakdown* of the strategies associated with secure, avoidant, and resistant/ambivalent attachment patterns (Hesse and Main, 2000). An impressive body of research has shown that disorganized attachment in a child is a reliable predictor of internalizing and externalizing disorders and diminished social competence during development (see, e.g., Fearon et al., 2010; Groh et al., 2014).

In contrast with what was found with respect to the parents of secure, avoidant, and resistant/ambivalent infants, Main and her colleagues were not able to identify additional generalized violations of coherence in the discourse of parents of disorganized infants (Bakkum et al., 2022a). Rather, what they found in these parents’ discourse were brief lapses of incoherence or confusion, which appeared specifically during discussions of potentially traumatic events, such as losing a relative or physical abuse. For example, Main and colleagues found that these parents expressed irrational ideas about death, or contradicted themselves, first by reporting having been abused, and then denying having experienced any abuse at all. Main and her colleagues expanded their AAI classification system to encompass these and similar markers as indicators of an unresolved (U/d) “state of mind with respect to attachment.”

Main and her colleagues have made two central claims about the psychological states associated with the U/d classification (Bakkum et al., 2022a). First, they proposed that the inability of U/d adults to coherently discuss their experiences of loss and abuse signals a tendency to enter dissociative, trance-like states. Second, they hypothesized that such abnormal processing occurs in response to fear that ensues when recalling traumatic attachment-related memories.

Following their own understanding of infant disorganized attachment, and their claims about the importance of brief dissociative episodes in U/d, Hesse and Main (2000) conceptualized the U/d classification as a disturbance in otherwise well-consolidated representational architectures. For this reason, they have defined the U/d state of mind not as a ‘fourth attachment pattern’, but rather as a temporary failure in the functioning of the regulatory mechanisms typical of the other classifications. This is why a U/d classification is assigned *in addition to* (and not in place of) the preoccupied, dismissing, and secure classifications. This view is well synthesized by the following passage:

In contrast [with the organized insecure attachment categories: autonomous/secure, dismissing, preoccupied] the intrusion of lapses of reasoning or discourse during the attempted discussion of potentially traumatic events that identifies unresolved speakers [during the AAI] does not appear to me to be part of an interactive pattern, or to represent a propensity toward a particular kind of relationship with the interviewer. The resemblance to infant Strange Situation behavior may then be said to consist in the fact that there has been an episode of disorganization [...] that represents not so much an overall pattern of interaction as a collapse of patterning (p. 443–444, Main, 1995).

Passages similar to this one are at the roots of the popular view of the U/d attachment classification as a localized, punctiform phenomenon. However, whilst the “lapses of reasoning or discourse” associated with U/d might not reflect a coherent orchestration of behavior, this does not mean that the interpersonal interactions of U/d speakers may lack other distinguishing features. In fact, a recent study of over 1,000 AAIs showed that U/d individuals show a distinctive pattern of physiological dysregulation throughout the entire AAI and not exclusively whilst discussing trauma or loss (Bakkum et al., 2022b). This suggests that it may be possible to identify distinctive interactive features of U/d speakers even outside of discussions of trauma or loss.

Moreover, some prominent attachment scholars such as Hesse (1996) and Lyons-Ruth and colleagues (e.g., Lyons-Ruth et al., 2005) have found evidence that there are individuals who are not secure, dismissing, or preoccupied, but rather demonstrate a pervasive and not localized discourse organization that can be detected *throughout* the AAI, beyond discussions of trauma and loss. For example, such a discourse organization can be revealed by profound and consistent violations of coherence, or by a tendency to tell narratives that are organized around feelings of fear. These cases have been termed by Hesse (1996) ‘Cannot Classify’ (CC, see also Speranza et al., 2017), and by Lyons-Ruth and colleagues “Helpless/Hostile” (2005). As far as the CC classification is concerned, Main and Hesse (1992) have suggested that individuals who received this classification may represent the far end of the continuum of the same process of disruption of processing manifested by briefer U/d markers. On the basis of this theory, as well as for reasons of statistical power, the convention has been for researchers to analyze CC and U/d together.

### Research on attachment-related differences in the psychotherapy process: Methods and results

In the past decade we have learnt more about the interactive and discursive processes linked with secure, dismissing, and preoccupied AAI classifications in psychotherapy sessions. The introduction and validation of the Patient Attachment Coding System (PACS) has shown that one can accurately predict patients’ AAI classifications by tracking aspects of patients’ discourse with attachment-related scales (i.e., Proximity-seeking, Avoidance, Resistance, etc.), regardless of whether patients talk about their parents or attachment figures (Talia et al., 2017, 2019a). Although this research has not focused on U/d, knowledge of its methodology and findings informed our search for differences related to U/d. We will thus discuss it briefly here.

Before its large-scale validation, the PACS was originally devised with a modified version of the guess and uncover method (see, e.g., Talia et al., 2014, 2017). Because the topics addressed in psychotherapy sessions are far more variable than those discussed in the AAI, in their early analysis Talia and his colleagues could not rely primarily on analyzing linguistic content or form. They embraced instead a *functional approach to discourse analysis*, a branch of discourse analysis that is committed to investigate “what language is used for” (p: 1, Brown and Yule, 1983). Language, to the extent that it is intended to affect an audience, serves a myriad of functions: informing others, giving commands, promising things, and so on (Wittgenstein, 1953). In the functional approach, the discourse analyst groups together and describes various types of utterances that seem to serve similar functions. By mapping how various functions occur in naturally-occurring conversations, the discourse analyst aims to reach a deeper understanding of the forces that animate talk exchanges (Brown and Yule, 1983).

**Figure 2:**
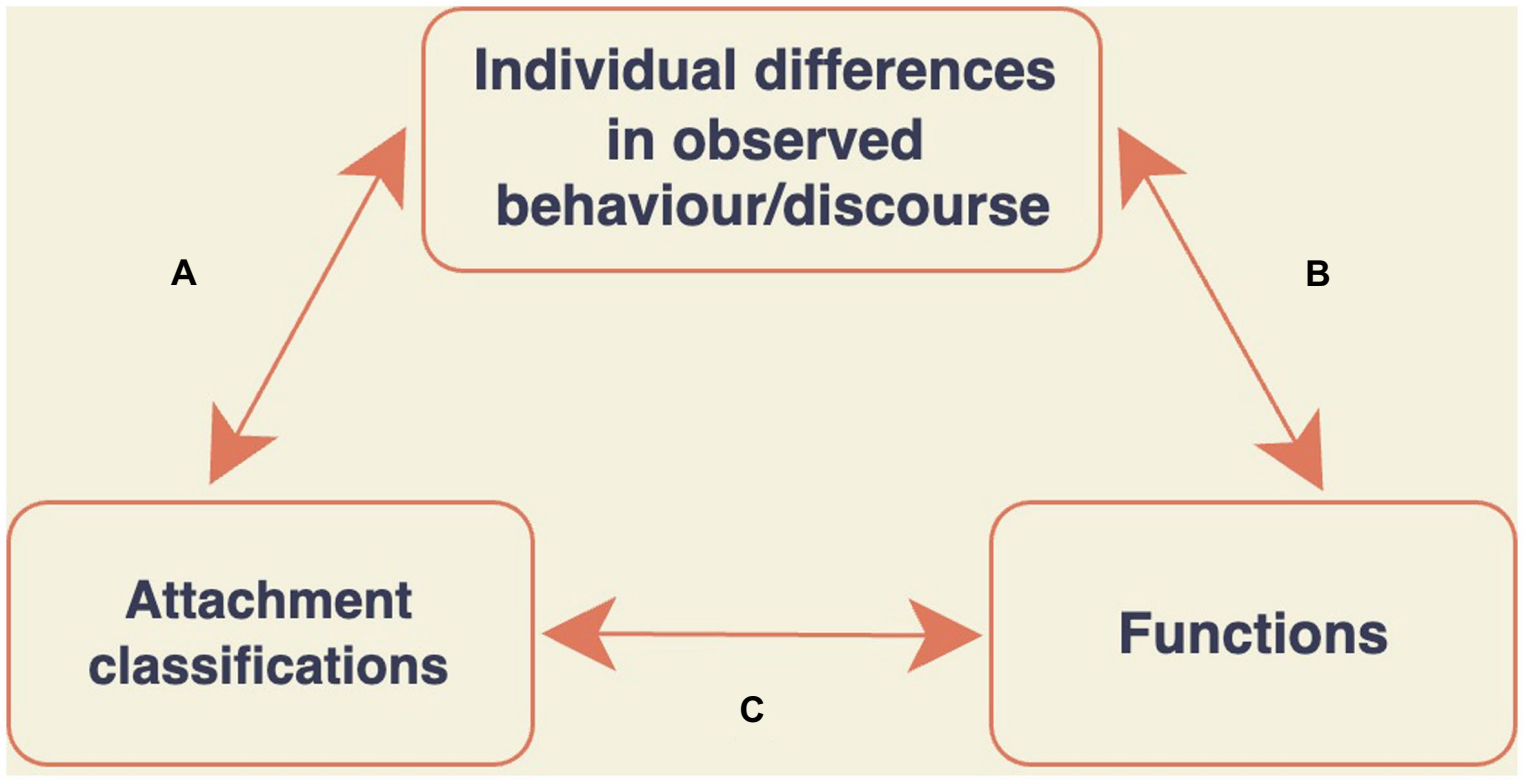
The guess and uncover method modified by Talia et al. (2014).

The addition of functional discourse analysis to the guess and uncover method allowed Talia and his colleagues to triangulate their observations of in-session discourse and behavior (see Figure 2). Similar to the traditional guess and uncover method, in this modified approach researchers try to establish correspondences between patterns of discourse or behavior and individuals’ independently obtained attachment classification (see *a,* Figure 2). At the same time, however, researchers also group utterances according to their probable function (see *b*, Figure 2). They do so by analyzing the likely effect of those utterances on the listener (e.g., increasing closeness and connection, avoiding disagreement), and, reciprocally, by using linguistic functions that seem to be common for identifying new utterance types (see Talia et al., 2014). Once an utterance is understood as predictably leading to a certain effect, it can be viewed as strategic or goal-targeted even if its effects are not consciously represented by the speaker (Talia et al., 2014). Although this particular approach to discourse analysis had not been used before by attachment researchers, it is consistent with the ambition of attachment theory to understand behaviors within broader patterns and interpret them in light of the functions they serve (see, e.g., Ainsworth et al., 1978).

Finally, following attachment theory, the functions hypothesized by the analyst in (b) in this method can be related to individual differences in attachment (c, Figure 2). As pointed out by Main (1981), different interpersonal expectations may call for different kinds of attachment-related behaviors. Knowledge of attachment patterns or classifications, which have been viewed as summarizing different interpersonal expectations, may thus provide rich suggestions about the possible functions that a certain attachment-related behavior may serve (Main et al., 1985). For example, infants’ avoidance of caregivers when alarmed seems at first paradoxical. However, avoidance becomes more comprehensible when it is understood as an attempt to attenuate demands for closeness, if the infant expects that the caregiver will rebuff such demands (Main, 1981).

With this method, Talia et al. established a taxonomy of utterance types associated with the different attachment classifications. They described two opposing discourse strategies, respectively, linked with dismissing and preoccupied attachment classifications. They also proposed that secure patients strike a balance between the two. In the course of their research, they examined various functions that might explain these various strategies, eventually focusing on how patients foster epistemic trust in the listener (Talia et al., 2019b).

Talia et al. found that dismissing patients, regardless of the topics discussed, tend to speak in a concise manner, relaying summaries and short explanations in place of narrative episodes and feelings. Because this way of communicating is simple to follow but may often appear as lifeless, Talia and his colleagues hypothesized that it may result from an attempt to keep communication as simple and succinct as possible, at the expense of making it interesting to the listener. They related these functions with dismissing patients’ unconscious expectation that interlocutors do not expect to acquire useful information from them, and that they will stop paying attention if the patient communicates in ways that require too much effort on the part of the listener to be followed (Talia et al., 2019b).

On the other hand, Talia et al., found that preoccupied patients tend to fill their discourse with direct quotations, long re-enactments of past episodes, and evocative but vague phrases. In this way, these patients command attention to their communication, but are more difficult to understand. From a functional perspective, they seem to strive to attract listeners’ interest, even if that means that a listener will not be able to attend to all they say. In turn, these functions seem to reflect an assumption that listeners will pay attention to their communication, but only inconsistently (Talia et al., 2019b).

In contrast with work exploring the influence of secure, dismissing, and preoccupied attachment on the therapy process, no empirical research has focused on how patients with a U/d attachment classification speak and interact with the therapist in any given session. This knowledge gap parallels a more general scarcity of research on the influence of the U/d attachment classification on the psychotherapy process and outcome (Slade, 2016). With the exception of a few studies suggesting that U/d attachment can be modified through psychotherapy (Levy et al., 2006; Buchheim et al., 2017; Tmej et al., 2018), our knowledge about the influence of U/d on therapy process and outcome is fairly limited (but see Reiner et al., 2016). This knowledge gap may be due in part to the widespread assumption that a U/d classification represents only the breakdown of “pattern” and “organization,” and therefore can have no distinguishing interactive features. Another reason for this diminished attention may lie in difficulties of administering the AAI in clinical settings. Interviews such as the AAI can typically be administered only once or twice during treatment, and at any time patients may decline to respond to questions about past trauma, which will effectively prevent coders from assigning a U/d classification.

Despite the absence of empirical research on this topic, there has been extensive, speculative discussion about how the U/d classification is expressed in in-session processes (Duschinsky and Foster, 2021). For example, Holmes (2001) and Wallin (2007) have argued that, in continuity with the way they speak in the AAI, U/d patients may be characterized in session by momentary dissociative lapses when discussing traumatic experiences. Further, following the finding that in the preschool years infant disorganized attachment becomes “organized” by the effort to control the caregiver (Main and Cassidy, 1988), Liotti has proposed that U/d patients are typified in session by their attempts to keep the therapist under control, either by attempting to humiliate the therapist, or by compulsively giving him/her attention and care (see Farina et al., 2020). Finally, Bateman and Fonagy (2016), and Johnson (2008) have proposed that U/d could manifest in clinical settings through contradictory strategies for seeking closeness with the therapist, for example by being alternately preoccupied with maintaining closeness, and then avoiding the therapist. The goal of our study was to expand and deepen these perspectives through actually observing U/d patients’ discourse during sessions of psychotherapy.

## Materials and methods

This article presents the results of analyses based on the modified guess and uncover method described in the previous section, in which we compared the discourse of 40 U/d patients in one single session against the discourse of patients who did not receive a U/d classification. Before treatment, all our participants had been interviewed with the AAI, and all, with the exclusion of four, had been interviewed with a validated diagnostic interview for assessing personality disorders. AAIs were transcribed verbatim and coded by reliable AAI coders. Though our focus in this paper is the analysis of the in-session discourse of U/d patients, we considered in the U/d group also patients who had received a “cannot classify” classification, because most CC patients also received a U/d classification, and because the two classifications have not so far been empirically or conceptually distinguished.

### Participants

The sample (N = 181) from which our participants were extracted included 20 patients (4 of which were classified as U/d or CC), who came from a counseling facility in Padua, Italy, where they received supportive psychotherapy; 72 patients (11 of which were classified as U/d or CC) who came from a treatment facility in New York, where they received brief relational therapy or CBT; and 68 patients (11 of which were classified as U/d), who came from a randomized controlled trial study with bulimic clients that took place in Copenhagen, Denmark (Poulsen et al., 2014), where clients received either psychoanalytic psychotherapy (PPT) or cognitive behavioral therapy-enhanced (CBT-E). Severe physical and psychiatric conditions that would interfere with treatment (e.g., psychosis or severe personality disorders) were exclusion criteria in all groups of participants. These outpatients have already been analyzed by Talia et al. (2017) in a study of in-session attachment that did not consider U/d.^1^

In the present study, in addition to these patients, we also included an additional 21 outpatients recruited from two departments specialized in the mentalization-based treatment of severe personality disorders, in the psychiatric clinics of Gentofte and Roskilde (Denmark). These additional participants were included in order to consider a less high-functioning sample of patients than that analyzed by Talia et al. (2017). 14 of these patients were classified as U/d or CC.

In the total sample, the mean age was 32.4, and 72.5% were female. 91% were of Caucasian origin. The mean level of education was 16.3 years (SD = 0.79). All patients had at least one psychiatric diagnosis, and 43% had a personality disorder diagnosis. Among U/d patients, the mean age was 34.2, 75% were female, and 94% were of Caucasian origin. The mean level of education was 15.9 years (SD = 1.0), and 86% had a personality disorder diagnosis (see Table 1). None of the previous differences between this group and the group of patients who were not U/d was significant, except for the rate of personality disorder. The AAI classifications, personality disorder diagnosis, and gender of our U/d participants are listed in Table 1.

**Table 1:** Contingency table illustrating the categories of indicators represented in patients.

				Subtype 1			Subtype 2		
		**PD**	**AAI**	**Cat. 1**	**Cat. 2**	**Cat. 3**	**Cat. 4**	**Cat. 5**	**Cat. 6**
1	Padua	NR	U/F	x					
2		NR	U/E				x		
3		NR	CC/Ds	x	x	x			
4		NR	U/E				x		x
*5*	New York	PPD	CC/Ds	x	x	x			
6		NOS	U/CC/D			x	x	x	
7		BPD	U/CC/E				x	x	x
*8*		NPD	U/E	x	x	x			
*9*		SPD	CC/Ds				x	x	
10		NOS	U/Ds			x			
11		APD	U/Ds		x	x			
*12*		APD	U/E	x		x			
*13*		HPD	U/E		x				
14		PPD	CC/E						x
*15*		NOS	U/E		x			x	
16	Copenhagen	“	U/E						
17		APD	U/Ds						
18		“	U/E						x
19		“	U/E	x		x			
20		BPD	U/CC/E	x	x	x			
21		BPD	U/E	x		x			
22		“	U/E				x	x	
23		APD	U/Ds						x
24		BPD	U/E				x	x	x
25		APD	CC/Ds				x		
26		“	U/Ds	x					
27	Gentofte/Roskilde	BPD	U/E				x	x	x
28		BPD	U/E				x	x	
29		BPD	U/E				x		
30		APD	U/E	x	x	x			
*31*		BPD	U/CC/E	x	x				
32		BPD	U/E			x			
33		BPD	U/CC/E	x		x			
*34*		BPD	U/CC/E	x	x				
*35*		BPD	U/E	x		x			
36		BPD	CC/E				x		x
*37*		BPD	U/E				x		
38		BPD	U/E				x	x	
39		BPD	U/E	x	x	x			
40		NOS	U/E				x	x	x

ID, Female-identifying patients are in bold. PD, Primary diagnosis of Personality Disorder (other PD diagnoses may be present). BPD, Borderline Personality Disorder; APD, Avoidant Personality Disorder; DPD, Dependent Personality Disorder; SPD, Schizoid Personality Disorder; PPD, Paranoid Personality Disorder; NOS, Not Otherwise Specified. AAI, attachment classification (primary/secondary, separated by a slash). F, secure; Ds: Dismissing; E, Preoccupied; U, Unresolved; CC, Cannot Classify. All the remaining columns (Cat.: category): “x” means the presence of at least one of the indicators belonging to the category of indicators described by that column.

### Procedure and data analysis

For each patient, we analyzed the third therapy session to ensure proximity to the administration of the pre-treatment AAI, or the nearest available session when the third one was unavailable. The sessions were transcribed following similar guidelines to those indicated for the AAI. All sessions were coded with the PACS and received a classification as secure, dismissing, or preoccupied. According to the PACS coding, no cases seemed to simultaneously display high avoidance and high resistance, including those who were classified as U/d in the AAI. In the group of U/d patients, the correlation between the PACS Avoidance scale and the PACS Resistance scale was *r* = −0.47, whilst in non-U/d patients was *r* = −0.50. This suggests that, at least among our participants, U/d may not be typified by simultaneous displays of avoidance and resistance.

Bi-weekly meetings were held by the first and the third author, who applied the modified guess and uncover method described above. In these analyses, they aimed to identify characteristics that may distinguish the 26 patients who had received a U/d classification in the sample studied by Talia et al. (2017, see footnote 1) against 26 patients from the same sample who were not so classified. The investigation began by searching for evidence of the U/d in-session characteristics hypothesized in the literature (i.e., dissociation, controlling behavior, simultaneous avoidance and resistance). Comparing two sessions at a time, this analysis resulted in a list of 30 discourse indicators that seemed to typify U/d patients.

This analysis was later expanded with the same method on the additional group of U/d patients from Gentofte and Roskilde, with the help of the fourth and fifth author. In eight successive rounds of group discussion, the indicators previously identified were applied to distinguish the new group of patients classified as U/d from a group of patients of equal size from Talia et al. (2017) study who were not so classified. While it was globally felt that the previously developed list of indicators also worked to distinguish U/d patients in this group, this new round of analyses served to refine the description of the indicators, as well as to group them into 6 categories according to their most salient feature. Furthermore, seven of the indicators previously proposed were excluded because they did not appear in this new group of participants. Two new indicators were added to the list after a re-analysis of the sessions of the rest of the sample confirmed that they were present also in the other subgroups of patients. Finally, it appeared that the indicators in the list could be divided in two subsets, because patients who tended to display indicators from one of these subsets did not display indicators from the other. In the absence of a theory that would explain the differences between these two subgroups of indicators, the patients primarily displaying one subset of markers or the other were tentatively classified as “Sub-Type 1’’ or “Sub-Type 2’’

## Results

In the following, we report evidence of six main categories of discourse indicators, which appeared to be associated with U/d or CC AAI classifications in our participants (see Table 1). Among our participants, there were at least 10 different patients who exemplified each category of indicators. We tentatively present these characteristics as falling under two hypothesized subtypes.

In addition to the indicators of a U/d classification that we discuss in the following, U/d patients demonstrated to an extreme degree the discourse characteristics that typify insecure speakers in the PACS. They either told narratives that were highly incoherent and confusing, or they told no narratives at all. Their description of interpersonal relationships was bidimensional and lacked integration. They almost never reported feelings, and even more rarely seemed to reflect about other people’s mental states. The interested reader is referred to the work of Talia et al. (2017, 2018) for additional information regarding in-session markers of insecure AAI classifications.

In our analyses, we focused on discourse characteristics that would emerge more broadly and not exclusively in relation to discussing loss and other potential traumatic events. Out of the 40 sessions/patients analyzed by us, only 9 contained discussion of potentially traumatic events, defined as any mention of any event in which the patient or any other person known to them did, or could have, lost their life or their physical integrity. Our choice was motivated by the hope of providing information that would be salient to clinicians and researchers even in those sessions when a patient does not discuss any traumatic event.

### Sub-Type 1: The patient does not encourage acceptance or endorsement from the therapist

#### Category 1: The patient makes statements that lack justification or seem contradictory

A notable feature of the in-session discourse of many patients classified as U/d was the absence of attempts to justify their own statements. Though insecure patients may sometimes be less effective at building belief in what they say than their secure counterparts (Talia et al., 2019b), they still seem to make some effort in fostering the listener’s trust in them. In the AAI, preoccupied speakers relay narratives that build their case and enlist the listener’s agreement. Dismissing speakers describe their parents in normalized, glib ways, which minimize the likelihood of disagreement. Many U/d patients were utterly different in this respect. In the discourse of these patients, it was common to find strong claims with no supporting evidence at all.

One scenario in which this characteristic was most conspicuous was when patients provided evaluations of others that were categorically negative. One patient said that his ex-wife “*was possessed, how can I say it, like there was a brain change, and things that were very unpleasant*,” without following the statement up with any illustrating memories or supporting accounts. Another patient dryly remarked: “*My ex-business partner was a bastard. Period*” and changed topic. Other patients attributed malevolent intentions to others, but without explaining why others might have had them. One patient bluntly said: “*Policemen are trying to trick me so they can send me back to jail*.” Another patient said: “*My mother wants me to look bad in the eyes of my daughter.”* In both cases, patients did not try to explain what feelings or needs might motivate policemen’s or their mother’s malevolent intentions.

The following transcript excerpt from another patient provides further illustration of both these discourse features:

*(1) Patient: After I moved home back from Italy, I met another man. But uh it didn’t really come to anything, because at the time I had begun living with my brother Joseph and he was so dismissive of him. And that man was doing some nasty stuff.*

***Therapist: So uh how nasty stuff?***

*Patient: Well, he made some threatening calls to my brother and everything.*

In this passage, the patient defines his brother as “dismissive,” the man as doing “nasty stuff,” and the calls as “threatening,” without any illustration or explanation. Immediately after, when the therapist probes the patient to say about such attributions, the patient fails to answer:

***T: What was it like for you, because/***

*P: /You think at first that it can’t be true, it’s my brother who’s wrong, isn’t it? Well, you can get a bit blind in a situation like that. But it was true*.

***T: And why did he make those threats to Joseph?***

*P: Yep, he did. No, I don’t know. I never asked him. Never*.

There are other ways in which U/d patients may come across as difficult to believe. For instance, many of these patients made assertions that seemed to contradict each other, without taking stock of the contradiction, as in the following example in which a patient talks about her girlfriend:

*(2) Well, she’s not going to keep thinking this is cool. Or something. So I feel like our relationship is a ticking time bomb. But I don’t really think it is. But I feel. Yeah. I can’t actually remember what the question actually was.*

A similar tendency could be found in situations in which a patient simultaneously affirmed and denied that some or other experience had occurred. For instance:

*(3) But I feel fine with that and I haven’t puked… well actually I have but…I have eaten well. I ate all that candy yesterday but I think that, I think that I’m eating really well, but I had a whole pizza and some candy so actually I think it’s a bit too much!*

Other U/d patients shared unusual values or systems of beliefs, again without explicitly defending or justifying them to the therapist, or they seemed to blur the distinction between fantasy and reality altogether. For example, one patient began telling the therapist about her dream to become a famous musician and own a private jet to travel across Europe. During several minutes, she then became so involved in describing the details of her (imagined) travels that she then started to omit the fact that what she was describing was a fantasy.

#### Category 2: The patient does not demonstrate empathy for others

Many patients in our sample often conveyed contempt or disinterest for other people’s experiences and feelings, for example, by laughing about others’ suffering, by belittling the worth of others as persons (“*My ex was just a total psychopath*”), or by failing to express any compassion about others’ possible fear or pain.^2^ See, for example, the following passage:

*(4) At that time my grandmother had gained a lot of weight, and her mind was starting to go, and my dad was taking care of her. One day he just couldn’t take it anymore…he was pushing her wheelchair down the street and she was moaning a lot, being critical. Well, that time my father couldn’t take it anymore and suddenly he threw her into the passing cars. She was very, very old, and he had just gotten so mad at that moment. I thought that only a good person who loved my grandmother so much could do that. I know it’s paradoxical, but at that moment I thought that.*

In other cases, these patients showed no self-consciousness about actions of theirs that may have negative impacts on others, as in the following example:

*(5) I called her this morning and asked her what she* [note: the patient’s girlfriend] *had done on Friday. Because I felt she’d been acting strange since she’d been at that party. I was very strong with her and said, “You know what? You know what, I’m going to have you tracked.” So we’ll see.*

Passages similar to these could be taken as evidence of a specific deficit in these patients’ capacity to think about other people’s mental states, or *mentalizing*. However, we found many instances in which patients not only disregarded other people’s feelings, but explicitly presented themselves as individuals who do not care about others, who are untrustworthy, or even dangerous. For instance:

*(6) So I was thinking about my sister and um she’s – she’s – to the best of my knowledge she’s still alive – I have not heard from her in over fifteen years um.*

*(7) I’m used to having boyfriends who are much older than me. But uh ... I’m also used to having someone I can manipulate - and kind of bully a bit. I need to bully people.*

If one wants to encourage the listener to endorse what one says, not only is it important to appear as a credible informant. It also seems important to appear as a cooperative individual who is attuned to others and their experiences. By showing no empathy or caring for others, the patients in this section may make the therapist less inclined to endorse their views or offer them sympathy. Thus, similarly to the examples in the previous section, these patients’ discourse seems to forgo the possibility of eliciting the therapist’s endorsement and acceptance.

#### Category 3: The patient seems to discount the possibility of influencing or being influenced by the therapist

Almost all of the patients of this subtype bluntly refused to respond to the therapist at least once in their session, indicating in this way that they did not expect communication with the therapist to be relevant to either them or the therapist. For example:

***(8) T: I’d like to try to understand what thoughts came up as you were feeling depressed last week when I had to cancel our appointment.***

*P: Nothing. It’s not related to you going away at all.*

***(9) T: What was going on inside of you that made you do that?***

*P: Mmm, no, I’m not going to answer that.*

These negative expectations about communication were made explicit whenever these U/d patients made flippant, humiliating comments about the therapist, for example saying that psychotherapy was not a serious profession, that the therapist was too young to understand the patient, that the therapist’s interventions were too predictable (e.g., “*I know a therapist would just say that!”*), or that speaking about emotions “*is not like difficult, like a problem of engineering or something*.”

These patients also seemed to attempt to gain control of the interaction with the therapist in other ways than through open communication about their own experiences. For example, they gave direct instructions or commands to the therapist, or they demonstrated an insistent and overbright attention towards the therapist. Some of these patients would open a session by asking the therapist about their holidays, showing concern about the therapist’s health, or they made direct and personal comments on the therapist’s appearance, as in the following examples:

*(10) P: Did you go on holiday? And how was your vacation?*

*T: It was nice and relaxing, thank you.*

*P: That’s always good. That’s the point right? Did you go away?*

*T: Ahm. I did.*

*P: Out of the States?*

*T: Yeah.*

*P: Good. Very good for you.*

*(11) T: And what did you see in your teacher’s eyes?*

*P: . . . . . {{5 sec}} your eyes are beautiful.*

*T: Hmm. Ok, wait a second we can talk about this later…What did you see in your teacher’s eyes? Perhaps a thought came about what she was thinking of you?*

*P: You’re very attractive.*

Finally, we often observed that these patients seemed to closely track the therapist’s mental state, perhaps again in an effort to control them. The following comments occur in the third session in therapy of three different patients:

*(12) T: I’m trying to understand what you’re asking. um*

*P: I think you know what I’m asking. I’ve made that clear.*

*(13) T: What sorts of thoughts do you wonder are correct or incorrect?*

*P: Are you trying to get me to think, to think, to bring it out, to bring it out myself –is that what you’re trying to do?*

*(14) T: So, you are left now to kind of have these experiences and deal with them…entirely on your own.*

*P: Well um for the most part, for the most part yes. What about you [name of therapist], do you share your uh bad days and negative emotions with your uh loved ones and those you are close to?*

### Summary of subtype 1

In this section, we illustrated three main features that emerged in the discourses of the U/d patients analyzed by us. By neglecting to provide evidence for what they are saying and by giving up on maintaining internal coherence, these patients seem to forsake seeking the therapist’s agreement. Similarly, by presenting themselves as untrustworthy individuals, they seem to provide little reason for the therapist to endorse what they say. Finally, by showing that they are not interested in what the therapist has to say about them, and by attempting to control the interaction through seemingly hostile behaviors or role-reversal, these patients discourage communication and connection.

### Subtype 2: The patient omits “the point” they are trying to make through their communication

#### Category 4: The patient reports impactful interpersonal experiences without elaborating on their evaluations and feelings

We have discussed how some of the U/d and “cannot classify” patients we analyzed appeared to make no effort to convince the therapist to accept their point of view. We will now consider the discourse style of another group of such patients, who were characterized by another, seemingly opposing discourse feature. With these patients, there will hardly be any doubt that their communication can be accepted as true. The difficulty with them seems to lie in identifying what their point of view on the topic under discussion is.

A striking example of this characteristic is offered by patients who fail entirely to mention their past or present reactions to the specific events they report. This lack is particularly remarkable when the patient talks about events that must have had a strong emotional impact on them. For example, a patient in our sample recounted being left alone by her best friend in the middle of a concert, and another patient told about being beaten up by a stranger in the street. Neither followed up their recounting with any judgment about what occurred or mention of how they felt at the time or now, nor did they seem to downplay their distress or involve the therapist in it. See for example the following excerpt:

*(15) P: And whatever so then I was with a guy for 3 years and…well I left some things out in fact.*

*T: Out of the story you mean?*

*P: Yes. I was raped by my cousin in a car park when I was 16* (the patient remains silent and then changes topic).

In other cases, patients talked about less extreme experiences, but which were difficult to interpret in the absence of any evaluation or feeling volunteered by the patient in relation to them. For example, one patient said:

*(16) I hate eggs and my wife always makes me eggs. She can’t fathom that someone may not like eggs. And still today I don’t understand why she keeps cooking me eggs.*

Similarly, other patients reported an emotional or behavioral problem, such as an inability to form close relationships or a tendency to angry outbursts, without saying how these problems made them feel. They did not ask for help with respect to such problems, they did not criticize themselves for having them, and they did not downplay them. The remarks are expressed as if the problems were effectively experienced by another. For instance:

*(17) I doubt if I’ll ever go off these meds. I don’t think the chemistry in my brain is going to change. And, you know, there’s obviously a lack of serotonin flowing around there somewhere and, you know. People have high cholesterol and people have, uh, other diseases and I have a lack of serotonin, and that’s it. And, uh, you know, I don’t know what brought it on.*

Finally, some of these patients glossed over their interpersonal experiences in ways that were so blatantly contradictory that they effectively left the chronicle of their experience without any evaluation, as in the following example:

*(18) I didn’t have any relationships at all with my parents. But I mean, I don’t have bad memories of my parents, I have good memories.*

In all these examples, without a clear statement by the patient about what he or she thinks about the experiences reported, the therapist is left, so to speak, alone to determine what exactly the patient is trying to communicate to him or her.

#### Category 5: The patient reports events and experiences only incompletely

Another discourse characteristic that recurred in the U/d patients we analyzed was their tendency to report experiences without recounting how they ended, almost as if they found it difficult to maintain their attention on completing the narratives they are telling. One patient vividly portrayed a harrowing episode in which she hid in a closet whilst her mother had come home drunk and threatened to beat her up. Yet the patient apparently forgot to mention whether she eventually had been beaten up or not in that instance. Another patient told the therapist about a bike accident where he injured himself and started to bleed copiously. Yet again this patient did not mention how the injury was taken care of afterwards. The next example illustrates a similar type of discourse:

*(19) It’s September 1983. I’m waiting to board the Stratford school bus. Several kids look at me and a girl throws a dirty tissue at me. Another girl to my right starts to kick my bum and a group of them form a circle around me throwing pebbles at me. Then there is an older kid who joins them and begins to hit me with a heavy stick. Nobody comes to help. I think that I am defective and probably not human. I am ugly, fat, and useless. I wanna disappear but I probably can’t even manage that. I’ll never have any friends and I’ll fail at anything I try.*

Other patients in this group mentioned future situations in which they or their significant others may be afraid or in danger, without explaining how they intend to soothe the fear or avoid the danger:

*(20) T: So you are saying it’s not true, you don’t, there’s no reason to think that you would face the same kind of challenges with this job?*

*P: No, cos’ I have the skills, I have done it many times before…so it’s not that I don’t have the skills, it’s that. It’s just, I’m afraid.*

*(21) T: What do you think will happen when your child is born?*

*P: Oh I don’t know, I will probably, the world will probably end. Um I have a soft heart so I probably will not be good with that. I will probably die.*

#### Category 6: The patient does not respond to the therapist’s exploratory questions

A third characteristic of this second subgroup of U/d patients was their repeated failure to respond to simple therapist’s interventions. This characteristic is not wholly dissimilar from insecure patients. Without intending to, insecure patients sometimes respond incompletely to the therapist’s probing, either because their responses are too brief and provide insufficient details, or because they do not seem to provide clear or relevant answers to the therapist’s question (Talia et al., 2017). U/d patients’ failure to respond is more extreme. These patients often react to apparently simple exploratory questions, in which the therapist asks the patient to say anything that is on their mind, with prolonged silences or with profound incoherence:

*(22) T: Do you think you learnt anything from this experience?*

*P: . . . . . . . . . . . (pause, 00:11) Sorry, can you repeat the question?*

*T: Just if you think there is something you have taken away from this experience, of getting the job you wanted.*

*P: Mmm…(pause, 00:14). I don’t know what to say.*

*(23) T: Anything in particular you’d like to talk about today?*

*P: I didn’t write anything down but I did think about it, um there is this part of me that has sort of sums things up which I had done before I met you…and it becomes…I’m not sure if it’s a truth to me…the way I’ve summed up my babyhood and childhood and I’m…maybe that could be the second thing we discuss. When I encapsulate something in a clear cut idea of it… whether that’s helpful, which I believe it is, but also whether it holds me back, cuz I stay frozen.*

### Summary of subtype 2

In this section, we illustrated a second group of features that emerged in the discourses of the U/d patients in our sample. In the indicators grouped in category 4, the patient presents a series of facts without mentioning what they thought about these facts, or what emotions were aroused in them as a consequence. In category 5, the patient presents some incident that occurred to them by omitting its outcome. In category 6, the patient fails to respond to questions from the therapist that require them to say what they think. All three of these features seem to reflect a way of presenting information neutrally, without superimposing subjective perspectives that may distort the chronicle of what happened, or may constrain the interpretation of their narratives by a predefined end-point. It seems to us that the tendency not to make explicit what they intend to communicate, i.e., their “point,” can account for all three discourse features.

## Discussion

In this article, we described in-session discourse features that typified patients who had received a U/d or CC classification on the AAI. Specifically, we discussed evidence of six categories of discourse indicators, tentatively grouped in two subtypes. Patients of the first subtype seemed to make statements that appeared to lack justification; they neglected to present themselves as reliable or trustworthy; and they often seemed to convey that they did not expect communication with the therapist to be relevant. Overall, they did not seem to enhance the likelihood that the therapist would believe them or accept what they said. Patients of the second subtype seemed not to elaborate on the personal meaning of their experiences; they described episodes without reporting how they ended; and they occasionally displayed a marked inability to respond to simple therapist’s interventions. Overall, patients of this second group did not make clear what they had in mind and what was the point they intended to communicate.

Our results suggest that an expansion of the current conceptualization of the U/d classification may be in order, beyond the focus on isolated and trauma-related instances of incoherence. Our findings also present the possibility that the markers we identified are not specific to the treatment situation but are perhaps more general features of the way in which U/d individuals engage in conversation. For example, some of the markers of a U/d state of mind in the AAI, such as making psychologically confused statements in relation to loss or affirming and then denying having been abused, could be found to be localized examples of a more general tendency not to orient communication around enhancing listener’s belief and acceptance. If this hypothesis was confirmed, the indicators identified in this paper could be assessed during structured interviews such as the AAI, thus overcoming the limitations of existing assessments of U/d (chiefly, their reliance on whether traumatic events are adequately probed by the interviewer, p: 320, Duschinsky, 2020).

We would be remiss not to mention some limitations of our study. For the purposes of making novel observations, the strengths of the guess and uncover method must be acknowledged. Yet this approach has several limitations. First, its methodological assumption of a 1 = 1 correspondence between observation and criterion measure (in our case, patients’ AAI classification) risks overfitting a coding system to the data. As a consequence, some of the indicators of U/d discussed in this paper may be more characteristic of our participants in particular than of U/d patients in general. Second, given that variation in U/d states of mind is probably more consistent with a dimensional, rather than a categorical distribution (Haltigan et al., 2014) our decision to exclude any indicator from our list should they be found in a non-U/d transcript may have resulted in our list not being comprehensive. Third, the guess and uncover method cannot exclude that the differences identified in patients’ discourse may be also related to other variables that are closely associated with our criterion validity measure (e.g., mentalizing). Replication efforts of our observations, with coders blind to participants’ AAI classification and rigorous hypothesis testing are urgently needed.

It is important to consider that, given the high prevalence of patients with personality disorders among our participants, the discourse characteristics we identified may be linked with disordered personality functioning, and not only with a U/d classification. Given that the U/d classification is over-represented in patients with personality disorder (Bakermans-Kranenburg and van Jzendoorn, 2009), this may not be a genuine limitation. On the contrary, our indicators may reflect the disruption of functional mechanisms implicated in both U/d and in various personality disorders. For instance, the concept of “identity diffusion,” commonly linked with low levels of personality functioning, is used to capture failures to integrate good and bad aspects of a relationship (Kernberg, 2006). We believe that such failures may be conspicuous in many of the U/d indicators of “Sub-type 1,” where patients speak about others in resolutely negative and/or unintegrated ways, bereft of curiosity or compassion. As another example, it is often proposed that patients with severe personality disorder tend to elicit in others feelings that are similar to those they are experiencing (i.e., “projective identification,” Ogden, 1979). The tendency of “sub-type 2 patients” to discuss frightening topics without mentioning how they feel about them may covertly transmit a sense of dread to the therapist, which the therapist may feel as their own and not the patient’s.

Our observations present convergences and divergences with previous theoretical hypotheses about how U/d patients speak and behave in therapy sessions. Most U/d participants demonstrated a degree of disconnection between mental contents that were suggestive of dissociation, either because they seemed to have no access to information to support their blanket negative statements (see subtype 1), or because their emotional responses were flattened and they lost track of the therapist’s questions (see subtype 2). However, unlike in Main and Hesse’s hypotheses, our observations suggest that these apparent instances of dissociation did not seem to be specific to discussions of traumatic- or even attachment-related topics.

Secondly, the communication of many U/d patients seemed to have the effect of controlling the interaction with the therapist, which brings to mind Liotti’s suggestions that U/d patients may display controlling behavior in session. In this regard, it is interesting that analogues of controlling-punitive behavior (e.g., giving instruction to the therapist), and controlling-caregiving behavior (e.g., being overly preoccupied with the therapist) were displayed by patients belonging to the same subtype and sometimes by the same patient.

Finally, we found no evidence that U/d or CC patients engage in opposing strategies of avoidance and resistance in sessions. Further research is needed to ascertain whether this may be a consequence of our methods, or a genuine case of not finding the phenomenon in the data.

It is not yet clear what mechanisms might underpin the communicative characteristics of U/d patients discussed in this paper. It is our view that a focus on epistemic trust may offer a unitary framework to account for the apparently distinct linguistic indicators of U/d in session. We had previously established that, in psychotherapy, patients with an insecure attachment classification may appear to be less competent at fostering the therapist’s epistemic trust in what they say than their secure counterparts (see Talia et al., 2019b). By presenting information that is difficult to believe, or narratives whose point is difficult to understand, patients classified as U/d or CC seem to invite their listeners to an even lower epistemic trust. The observations of the in-session discourse of U/d patients seem to extend the view of Talia et al. (2021b), who theorized that attachment classifications capture ways in which individuals attempt to foster epistemic trust in their listener.

One possible interpretation of the two opposing subtypes identified in our sample can be derived from contemporary linguistic pragmatics. There is a broad consensus in the field of linguistic pragmatics that explicit and overt communication in humans can be seen as the result of two related but distinct intentions (see, e.g., Grice, 1957; Sperber and Wilson, 1986/1995). The first of these intentions, which can be termed *informative intention*, is the intention to change listeners’ representation of the world and thus to *inform* them. It reflects a desire to be believed. The second intention, which can be termed *communicative intention,* is the intention for the informative intention to be recognized. This is an intention to be understood, that is, to clarify one’s “point,” above and beyond the desire for that “point” to be accepted. Human communication is generally geared to fulfill both intentions (Sperber and Wilson, 1986/1995).

Against this backdrop, we would like to propose that the two subtypes of U/d patients described in this paper may be driven by one of these intentions at the exclusion of the other. U/d patients of the first subtype do not seem to trust that informative intentions can ever be successful. They may not expect to be believed. They may focus instead on getting their communicative intention fulfilled by clarifying what they intend to communicate, so that the listener will be able to decide by themselves whether to accept it or not at a later time. On the other hand, patients of the second subtype do not seem to trust that communicative intention can ever be relevant. They may expect listeners not to be interested in the points they want to make. They seem to focus instead on getting their informative intention fulfilled by presenting their experience in a bare, neutral manner, which can presumably have a strong impact on the therapist, albeit in a way that cannot be predicted in advance by the patient. Thanks to this study, these preliminary hypotheses could be elaborated upon in greater depth in future works.

This study is a first necessary step for future empirical investigations of the in-session discourse of U/d patients. Though our observations need independent validation, an observer-based measure of U/d attachment classification based on coding the frequency of these markers in transcripts may offer a more economic assessment than extant approaches for assessing U/d attachment in the clinical context. Such a measure, in turn, could provide a window into these individuals’ interpersonal behavior and encourage research about how U/d attachment impacts the process and outcome of psychotherapy. For instance, the characteristics we identified in the discourse of U/d patients may exert an influence on therapists’ feelings, on the therapeutic alliance, as well as on treatment dropout and outcome (Miller-Bottome et al., 2019). Finally, despite evidence that U/d attachment can change in therapy, no research exists about how such change may occur. An observer-based measure of this attachment classification, which tracks moment-to-moment discourse characteristics such as those discussed in this paper, may help us address this gap.

## Data availability statement

The datasets presented in this article are not readily available because the original interview data contains personally sensitive information and could make patients identifiable. The list of discourse indicators identified in this study are available to researchers upon request. Requests to access the datasets should be directed to alessandrotaliapsy@gmail.com.

## Ethics statement

The studies involving human participants were reviewed and approved by Research Ethics Committee of the Capital Region of Denmark. The patients/participants provided their written informed consent to participate in this study.

## Author contributions

AT, MM-B, SDM, and AW conducted the qualitative analysis presented in this paper. AT elaborated the original theoretical proposal presented in the paper and wrote the first draft. All authors contributed to the article and approved the submitted version.

## Funding

This work was supported by the Wellcome Trust [218025/A/19/Z], and by the International Psychoanalytic Association (grant number 055).

## Conflict of interest

The authors declare that the research was conducted in the absence of any commercial or financial relationships that could be construed as a potential conflict of interest.

## Publisher’s note

All claims expressed in this article are solely those of the authors and do not necessarily represent those of their affiliated organizations, or those of the publisher, the editors and the reviewers. Any product that may be evaluated in this article, or claim that may be made by its manufacturer, is not guaranteed or endorsed by the publisher.

## Footnotes

^1^The present study also include the analysis of four patients who had been excluded by Talia et al. (2017) because their AAI had been judged to be of insufficient audio quality.

^2^Readers familiar with the AAI may be reminded by these examples of a discourse feature initially associated by Main and colleagues to dismissing attachment classifications: derogation. Research has subsequently shown that this rare discursive feature is statistically related with unresolved states of mind, not dismissing attachment (Haltigan et al., 2014).
